# Propensity-score-matching-based analysis of laparoscopic gastrectomy with neoadjuvant chemotherapy for gastric carcinoma

**DOI:** 10.20407/fmj.2020-007

**Published:** 2020-10-10

**Authors:** Shimpei Furuta, Ichiro Uyama, Zenichi Morise

**Affiliations:** Department of Surgery, Fujita Health University, School of Medicine, Toyoake, Aichi, Japan

**Keywords:** Gastric cancer, Gastrectomy, Laparoscopy, Drug therapy, Morbidity

## Abstract

**Objectives:**

Neoadjuvant chemotherapy (NAC) is widely accepted as a potential treatment for advanced gastric cancer (AGC). Laparoscopic gastrectomy (LG) has recently been performed for advanced gastric cancer and could lead to improved adherence to multimodal treatment. In the present study, we compared the feasibility and outcomes of LG in patients with or without NAC in our institution.

**Methods:**

We assessed patients who underwent LG with (n=185) or without (n=1204) NAC between 1997 and 2013. We used propensity score matching to evaluate perioperative short-term outcomes and long-term outcome.

**Results:**

We used propensity score matching by patient background and treatment-rerated factors to establish two groups of 157 patients with or without NAC. There were no significant differences in perioperative short-term outcomes or long-term outcome between the groups.

**Conclusions:**

LG for selected patients with NAC is feasible and safe but has no long-term survival benefit.

## Introduction

Gastric cancer (GC) is one of the most frequent cancers,^1^ and surgical resection is performed in the early stage. However, surgery alone for advanced gastric cancer (AGC) has a limited beneficial effect on long-term outcomes.^2^ Therefore, multimodal approaches to treatment of AGC have been tried to improve patients’ survival. In the last 20 years, large randomized trials have demonstrated the efficacy of adjuvant chemoradiotherapy (INT-0116 trial),^3^ adjuvant single-drug chemotherapy (ACTS-GC trial),^4^ and perioperative three-drug combination chemotherapy (MAGIC trial).^5^ After publication of the results of these trials, surgery alone was no longer considered to be the standard treatment for AGC. Adjuvant chemotherapy after D2 lymphadenectomy is currently considered the standard treatment for GC.^3,5^ However, the prognosis for AGC remains poor compared with that of early-stage GC, with 5-year survival rates >95%,^6^ and there is no established method to increase survival.^6^ Neoadjuvant chemotherapy (NAC) might improve the prognosis of AGC because it can reduce tumor size, decrease clinical stage, and increase curative resection rate.^5^ S-1 (TS-1^®^; Taiho Pharmaceutical Company, Tokyo, Japan), which is a promising oral anticancer drug for GC,^7,8^ plus cisplatin therapy had a 54% response rate for AGC in a phase III trial. Although there were no treatment-related deaths in this trial, many grade 3 or 4 adverse events were reported. Severe adverse events in the NAC setting could lead to incomplete treatment or delayed surgery, and the ideal timing of surgery may be missed. Therefore, compared with laparotomy, laparoscopic gastrectomy (LG) is expected to improve adherence to multimodal treatment.^9–11^

In the present study, we evaluated the feasibility and outcomes of LG following NAC by propensity score matching (PSM)-based comparison of patients with or without NAC in our institution.

## Methods

### Study design

This was a single-center retrospective cohort study of patients who underwent LG for GC between 1997 and 2013 at Fujita Health University, Toyoake, Japan. We divided the patients into two groups: 185 with NAC and 1204 without NAC. We collected the following background data on the patients: sex, age, American Society of Anesthesiologists (ASA) status, body mass index (BMI), and clinical stage according to Japanese Classification of Gastric Carcinoma (JCGC) before treatment. We also collected data on the following treatment-related factors: surgical procedures (proximal gastrectomy, distal gastrectomy, total gastrectomy or pancreaticoduodenectomy); extent of lymphadenectomy (D1 plus or less, or D2 or more); combined resection of other organs; adjuvant chemotherapy; perioperative short-term outcomes (total operation time, estimated blood loss, postoperative hospital stay, and complication rate); and long-term outcome [Kaplan–Meier overall survival (OS) after surgery].^12^

**Figure 1:**
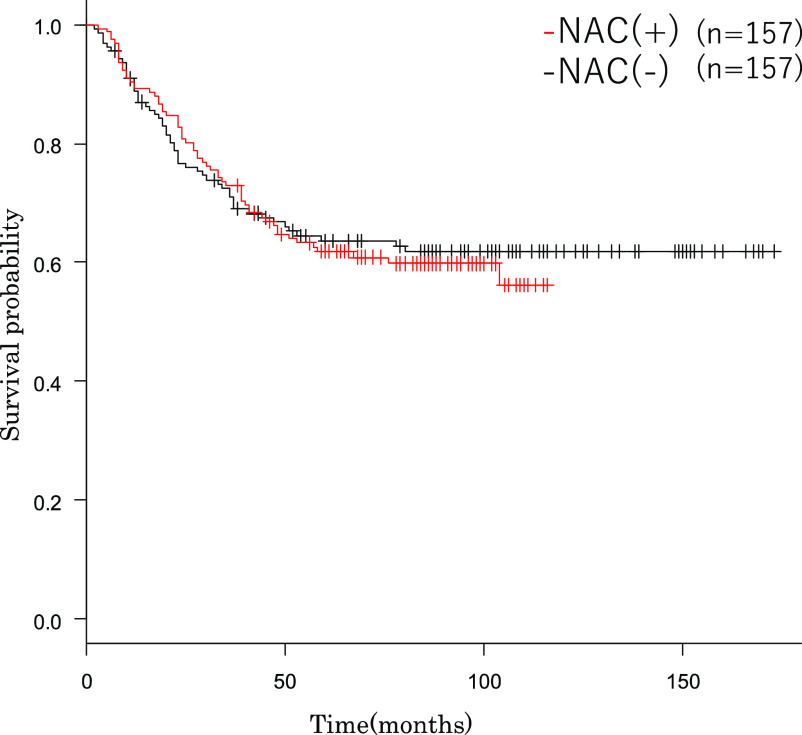
Survival curve A comparison of neoadjuvant chemotherapy (+) versus neoadjuvant chemotherapy (–) in advanced gastric cancer: A propensity score matching analysis NAC, neoadjuvant chemotherapy.

We compared these factors between the NAC(–) and NAC(+) groups (Tables 1 and 2). PSM analysis under the probability of 0.05 was used to limit confounders and overcome possible patient selection bias. After PSM, 157 patients were included in each group. The factors used for PSM were patient background (sex, age, ASA status, BMI and JCGC clinical stage) and treatment-related factors (surgical procedure, extent of lymphadenectomy, combined resection of other organs, and adjuvant chemotherapy). We compared patient background, treatment-related factors, perioperative short-term outcomes and long-term outcomes (Tables 3 and 4 and Figure 1).

The data obtained through review of medical records were managed according to the privacy policy and ethical code of our institution.

### Surgical procedure

The techniques and perioperative management of LG have been reported previously.^9–11^ Distal gastrectomy was used for tumors that were localized to middle and/or lower areas, whereas total gastrectomy was used for tumors that infiltrated the upper, middle and lower areas. Distal and total gastrectomy were both performed with lymphadenectomy. Proximal gastrectomy was used for tumors that were localized in upper areas. Pancreaticoduodenectomy was performed when the tumor invaded the pancreas.^13^

### Terminology

The GC stage was described according to JCGC, 3rd English edition.^14^ GC stage was determined by contrast-enhanced computed tomography, gastrography, endoscopy, and endosonography. Postoperative complications were defined as those that required surgical, endoscopic or radiological intervention, and that corresponded to Clavien–Dindo classification grade III or higher.^15^

### Statistical analysis

All analyses were conducted using IBM SPSS Statistics 23 (IBM, Armonk, NY, USA). Univariate analysis using the χ^2^ test was used for between-group comparison of numerical data, and the Mann–Whitney U test was performed for analysis of ordinal data (patient background, treatment-related factors and perioperative short-term outcomes). Data are expressed as median and range or odds ratio and 95% confidence interval, unless otherwise noted. A p value <0.05 (two-tailed) was considered statistically significant.

## Results

### Comparison with entire cohort (NAC(+) versus NAC(–))

Patient background and treatment-related factors of the entire cohort are shown in Table 1. There were no differences in ASA status and BMI between the NAC(+) and NAC(–) groups; however, there were significant differences in sex (p=0.036), age (p=0.004) and GC stage (p<0.001). Compared with the NAC(–) group, the NAC(+) group had more patients with total gastrectomy (p<0.001), greater extent of lymphadenectomy (p<0.001), more combined resection of other organs (p<0.001), and more adjuvant chemotherapy (p<0.001; Table 1).

The perioperative short-term outcomes before matching were shown in Table 2. Compared with the NAC(–) group, the NAC(+) group had significantly longer operation time (p<0.001), greater blood loss (p<0.001) and more complications (p<0.001). There was no significant difference in hospital stay between the two groups. There was no conversion to laparotomy in either group.

### Comparison with PSM cohort (NAC(+) versus NAC(–))

Two groups of 157 patients with or without NAC were established by PSM of patient background and treatment-related factors. There was no significant difference between the groups in patient background factors (sex, age, ASA, BMI or JCGC stage) or treatment-related factors (surgical procedure, extent of lymphadenectomy, combined resection of other organs, or adjuvant chemotherapy; Table 3).

Perioperative short-term and long-term outcomes after PSM were shown in Table 4 and Figure 1. There were no significant differences between the groups in perioperative short-term outcomes (total operation time, estimated blood loss, postoperative hospital stay, or complications; Table 4). There was no significant difference in long-term outcome OS (p=0.686; Figure 1).

## Discussion

Radical excision is still thought to be the only cure for AGC at present. However, chemotherapy has been developed as standard treatment for unresectable or recurrent GC.^16,17^ To improve survival, it is now considered that adjuvant chemotherapy, such as S1^4^ and S1+cisplatin,^9^ is necessary after surgery for AGC. The ACTS-GC trial^4^ showed that pathological stage II patients, excluding T1 cases, with S1 adjuvant chemotherapy had significantly better 3-year survival than patients with surgery alone in 2007. Thereafter, 1-year S1 adjuvant chemotherapy came to be recommended as standard in JCGC guidelines.^5^ However, treatment of AGC has not yet achieved satisfactory results. Therefore, a combination strategy with addition of NAC to radical resection with or without adjuvant chemotherapy is now being investigated to try and improve survival after surgery.^18^

The selected treatment strategy was based on AGC (including more than T2 (The T2 tumor has grown into the muscularis propria, the muscle layer of the stomach)) and surgeons following discussion of the disease and treatment and obtaining informed consent. Gastrectomy for AGC after NAC is a demanding a precise procedure and can lead to higher incidence of postoperative morbidity.^19^ Therefore, we thought that LG could reduce complications in these patients. In our institution, we perform LG in AGC patients after NAC, to facilitate early application of postoperative chemotherapy and obtain the survival benefit of this less-invasive procedure. We showed a complete or partial response in 96 of 185 (51.2%) patients according to the Response Evaluation Criteria in Solid Tumors version 1.1, and we were able to perform the operation smoothly. We investigated patient background, treatment-related factors, perioperative short-term outcomes and long-term outcome in patients with or without NAC who underwent LG between 1997 and 2013. NAC mainly comprised S1+cisplatin, as in the SPIRITS trial,^9^ or S1 regimens, as in the ACTS-GC trial.^4^ We also examined the perioperative short-term outcomes and long-term outcome in patients with or without NAC after PSM. There were no significant differences in perioperative short-term outcomes between patients with or without NAC after PSM. This shows that LG after NAC had similar safety to LG without NAC in these selected patients at our institution. However, there were also no significant differences in long-term outcome (OS) between the groups after PSM. We failed to show any survival advantage of our strategy with LG after NAC.

The main limitation of the present study was that it was a single-center retrospective cohort study with a long study period. Our study had a wide range of cases, including stage I and stage IV GC. For patients with stage IV GC, we performed palliative resection. The aim of the present study was to perform LG in patients with NAC to minimize postoperative complications. Our ultimate goal is to start postoperative adjuvant chemotherapy as soon as possible and improve the prognosis of AGC. Nevertheless, this study showed that LG could be performed safely, even in patients with AGC and NAC.

## Conclusion

Although a further prospective study is needed to evaluate the long-term outcome of our treatment strategy for AGC, the present study shows that LG is feasible after NAC in patients with AGC .

## Figures and Tables

**Table1:** Background and treatment-related factors of all patients who underwent laparoscopic gastrectomy with or without NAC, before propensity score matching

Patient characteristics (n=1389)	NAC(–) (n=1204)	NAC(+) (n=185)	p value
Sex (male/female)	824/380	140/45	0.036*
Age (years)^a^	67 (28–92)	64 (31–86)	0.004*
ASA status (I/II/III/Ⅳ)	502/551/148/3	95/67/23/0	0.070
BMI (kg/m^2^)^a^	22.0 (13.4–32.1)	21.7 (14.5–38.0)	0.461
Clinical JCGC stage (I/II/III/Ⅳ)	989/132/49/34	29/77/55/24	<0.001*
Surgical procedure (LPG/LDG/LTG/LCG/LPD)	70/859/238/36/1	3/100/76/2/4	<0.001*
Extent of lymphadenectomy (D1 plus or less/D2 or more)	827/377	25/160	<0.001*
Combined resection of other organs (%)	142 (11.7)	61 (40.0)	<0.001*
Adjuvant chemotherapy (%)	191 (15.9)	131 (70.8)	<0.001*

χ^2^ test was used for between-group comparisons of sex, comorbidity and history of laparotomy. We used the Mann–Whitney U test for between-group comparisons of age, BMI, and clinical JCGC stage. * Statistically significant. ^a^ Data shown as median (range). ASA, American Society of Anesthesiologists; BMI, body mass index; JCGC, Japanese Classification of Gastric Cancer; LDG, laparoscopic distal gastrectomy; LPD, laparoscopic pancreaticoduodenectomy; LPG, laparoscopic proximal gastrectomy; LCG, laparoscopic completion gastrecomy; LTG, laparoscopic total gastrectomy; NAC, neoadjuvant chemotherapy.

**Table2:** Perioperative short-term outcomes in patients undergoing laparoscopic gastrectomy with or without NAC, before propensity score matching

Patient characteristics (n=1389)	NAC(–) (n=1204)	NAC(+) (n=185)	p value
Total operation time (min)^a^	314 (129–937)	385 (189–962)	<0.001*
Estimated blood loss (g)^a^	39.0 (0–2267)	75.0 (0–1660)	<0.001*
Hospital stay following surgery (days)^a^	14.0 (3–150)	16.0 (8–122)	0.007*
Complications rate (%)	109 (9.1)	33 (17.8)	0.001*

* Statistically significant. ^a^ Data shown as median (range). NAC, neoadjuvant chemotherapy.

**Table3:** Background and treatment-related factors of all patients who underwent LG with and without NAC, after propensity score matching

Patient characteristics	NAC(–) (n=157)	NAC(+) (n=157)	p value
Sex (male/female)	120/37	117/40	0.695
Age (years)^a^	64 (28–87)	64 (31–86)	0.439
ASA status (I/II/III/Ⅳ)	81/63/13/0	81/56/20/0	0.558
BMI (kg/m^2^)^a^	21.6 (13–28)	21.7 (15–32)	0.300
Clinical JCGC stage (I/II/III/Ⅳ)	52/48/34/23	29/69/41/18	0.251
Surgical procedure (LPG/LDG/LTG/LCG/LPD)	4/97/51/4/1	3/87/64/2/1	0.349
Extent of lymphadenectomy (D1 plus or less/D2 or more)	21/136	25/132	0.525
Combined resection of other organs (%)	47 (29.9)	50 (31.8)	0.715
Adjuvant chemotherapy (%)	99 (63.1)	105 (66.9)	0.479

χ^2^ test was used for between-group comparisons of sex, comorbidity and history of laparotomy. We used the Mann–Whitney U test for between-group comparisons of age, body mass index, and clinical JCGC stage. * Statistically significant. ^a^ Data shown as median (range). ASA, American Society of Anesthesiologists; BMI, body mass index; JCGC, Japanese Classification of Gastric Cancer; LDG, laparoscopic distal gastrectomy; LPD, laparoscopic pancreaticoduodenectomy; LPG, laparoscopic proximal gastrectomy; LCG, laparoscopic completion gastrecomy; LTG, laparoscopic total gastrectomy; NAC, neoadjuvant chemotherapy.

**Table4:** Surgical outcomes and short-term postoperative courses in patients undergoing laparoscopic gastrectomy with or without NAC, after propensity score matching

Patient characteristics	NAC(–) (n=157)	NAC(+) (n=157)	p value
Total operation time (min)^a^	398 (213–865)	372 (189–962)	0.207
Estimated blood loss (g)^a^	63 (0–2267)	75 (0–1514)	0.592
Hospital stay following surgery (days)^a^	16 (5–129)	16.0 (8–122)	0.399
Complications rate (%)	25 (15.9)	27 (17.2)	0.560

* Statistically significant. ^a^ Data shown as median (range). NAC, neoadjuvant chemotherapy.
